# Infertility grief and filiation experiences 25 years later: detection of multiple cases from the mixed methods approach

**DOI:** 10.3389/fpsyg.2025.1660802

**Published:** 2025-11-05

**Authors:** Núria Camps, Eulàlia Arias-Pujol, M. Teresa Anguera

**Affiliations:** ^1^Fundació Puigvert, Clinical Psychology Department, Barcelona, Spain; ^2^Eulàlia Arias-Pujol, Universitat Ramon Llull, Faculty of Psychology, Education and Sports Sciences Blanquerna, Research Group of Couple and Family (GRPF), Barcelona, Spain; ^3^Faculty of Psychology, Education and Sports Sciences Blanquerna, Barcelona, Spain; ^4^Faculty of Psychology, Institute of Neurosciences, Barcelona, Spain

**Keywords:** assited reproduction, infertily grief, origins disclosure, health psychology, mixed methods, multiples cases

## Abstract

**Introduction:**

The diagnosis of infertility generates a significant psychological impact on the couples who experience it. The loss of fertility triggers a psychological mourning process that affects self-esteem and identity. The aim of this study is the follow-up of couples 25 years after conceiving children with artificial insemination of donor semen, to explore from a mixed methods approach the relationship between the mode of elaboration of infertility grief, the communication strategies of the genetic origins of babies and the filiation experience.

**Method:**

The macrostages of the process are QUAL-QUANT-QUAL to integrate qualitative and quantitative elements. The indirect observational methodology has been used, based on in-depth interviews with infertile couples. The observational design is N/F/M: nomothetic (10 couples), incomplete intersessional follow-up (two time points) and incomplete intrasessional follow-up (single in-depth interview), and multidimensional. There are five dimensions proposed based on the theoretical psychoanalytic framework and the answers obtained, which are displayed in sub-dimensions up to the fourth level, and based on each of them a system of categories is developed. The observational instrument is *ad hoc*, and allows to systematize the transcriptions of the answers, obtaining matrixes of codes (249). After a process of transformation of the data, the quality control of the data is found, and a lag sequential analysis is carried out with the GSEQ5 software, with the aim of detecting regularities in the existence of behavioral patterns.

**Results:**

The total number of behavior patterns was 55. From the results of the lag sequential analysis five multiple cases have been detected that show that there is a connection between the conducts of the mode of elaboration of infertility grief and the communication of genetic origins and filiation experience.

**Discussion:**

Qualitative and quantitative elements have been integrated, which is innovative in this substantive area. Likewise, the rigor of a mixed methods approach has facilitated the identification of the deep hidden structure of nuanced aspects in grief coping analysis, which holds relevance for the psychological support of couples undergoing assisted reproduction.

## 1 Introduction

Assisted Reproduction Techniques (ART) at the 21st century have significantly increased the need for genetic material donation banks. Heterosexual couples, lesbians and single women use gamete donation to fulfill their desire to have a child (François and Bouychou, 2019).

The diagnosis of infertility involves great emotional pain widely studied in terms of anxiety, depression and coping strategies, as well as their relationship with pregnancy rates (Burgio et al., 2022; Flis-Trèves, 1998; Massarotti et al., 2019; Moreno-Rosset, 2000a, b; Muñoz et al., 2009; Oh and Shin, 2025; Rooney and Domar, 2018). It is also described from the clinical point of view as a “grief of griefs” composed of three nuclei of loss: (a) biological fertility, (b) the child project organized in psychoevolutionary development and (c) the asymmetry between one fertile and one non-fertile member of the couple (Camps, 1996, 2007). In male infertility, men have greater anxiety (Boivin, 2019; Hegyi et al., 2019; Öztekin et al., 2020; Patel et al., 2018; Sejbaek et al., 2020).

The elaboration of this complex grief is a vital crisis and behaves according to the organization of the personality, the relational dynamics established in the couple and their degree of vulnerability (Abdoli et al., 2025; Plowden et al., 2025; Espada and Moreno-Rosset, 2008; Camps, 1996, 2009, 2010a, b, 2018; Jeon et al., 2025; Muñoz et al., 2012; Assaysh-Öberg et al., 2023).

This research incorporates the assumption that grief coping strategies to face the loss of biological fertility (GCS) is related to the decision-making process of the communication of genetic origins to children and to the quality of the emotional bonds established during parenting.

The process of communication of genetic origins has been an issue widely discussed with voices for and against in which we observe very opposite positions. Some favor secrecy, but others favor communication (McGee et al., 2001; Schmid and Ehlert, 2025).

Many authors study the issue from the perspective of decision, timing, and how to communicate (Blake et al., 2010; Daniels and Thorn, 2001; Kirkman, 2003; MacDougall et al., 2007), reporting that biparental heterosexual couples present significant difficulties in making the decision.

Our clinical practice shows that when the indication of reproductive treatment with genetic donation is made, most couples have a tendency to refuse to recognize the difference between biological fertilization and assisted reproduction. At this time, they do not see the need to communicate genetic origins to their children and consequently it becomes a conflicting issue for the future (Camps, 2021).

Given the qualitative nature of the problem, the research adopted a rigorous study design from a mixed-methods perspective (Anguera, 2022). The initial phase focused on analysing habitual behavior within a natural context, specifically through in-depth interviews. These interviews were processed following the scientific method (Chacón-Moscoso et al., 2021), which involved: (1) building a non-standard instrument specifically adapted to the object of study, (2) systemizing the information, (3) controlling data quality, and (4) employing adequate analysis techniques, such as T-Patterns, to discover the deep, hidden structure within the data (Anguera et al., 2023a, b).

Interest in the content analysis of verbal data from in-depth interviews is growing rapidly in indirect observation research (Anguera et al., 2018), particularly within clinical and care settings (Arias-Pujol and Anguera, 2020; Del Giacco et al., 2020). However, this methodology has never been applied before in the field of ART.

The main objective of this research is to detect potential regularities in the modality of grief due to infertility (GCS) and patterns related to the communication process of genetic origins, based on an exploratory study of the natural history of infertile couples 25 years after having children with assisted reproduction techniques of donor semen (AID).

## 2 Methods

### 2.1 Design

Within the framework of the *mixed methods* approach, the observational methodology has been used, as it conforms to the essential requirements of a non-intrusive situation. Specifically, this is an indirect observation (Anguera et al., 2018), given that in-depth interviews are carried out, in which the interviewer and interviewees act horizontally. Indirect observation is especially appropriate when textual material analysis is required, and is recently being developed in different areas, such as in psychotherapies with adolescents (Arias-Pujol and Anguera, 2020) and with adults (Del Giacco et al., 2020).

The observational design is Nomothetic/Punctual/Multidimensional (N/P/M) (Sánchez-Algarra and Anguera, 2013): nomothetic because we studied 10 couples who had children born with AID; with intrasession follow up (in-depth interview), and multidimensional since the complexity of the aim required the application of various dimensions (that were included in the observation instrument).

### 2.2 Participants

The study included 10 couples, selected from a broader sample previously recruited in an earlier study (Camps, 1992). Although the selection was random within that pool, the number of participants was determined according to the principle of theoretical saturation, whereby data collection was concluded once no new relevant themes emerged. Couples were attended in the Assisted Reproduction Unit of the Puigvert Foundation (Andrology, Semiology and Embryology Laboratory, Clinical Psychology and Gynecology Service, Hospital Sant Pau in Barcelona, Spain) with a male factor diagnosis who conceived children through assisted reproduction techniques with donor semen. Of the 10 couples, 8 of them were attended as a couple (father and mother together), and 2 mothers came alone, since they had separated from their husbands. Although attempted, parents could not be reached. The ages of the total number of participants are between 45 and 60 years old. The mean age for men was 52.6 years (*M* = 52.6; range = 48–60), while for women it was 48.9 years (*M* = 48.9; range = 45–52).

All families reported a middle socioeconomic status, and their educational background corresponded to intermediate-level studies. Family composition was diverse: five couples had one child, four couples had two children, and one couple had more than two children.

In accordance with the principles of the Declaration of Helsinki the participants were informed that they were being recorded and they signed informed consent. The research was approved by the Clinical Research Ethics Committee (CEIC) under reference N/REF/2008/15.

Before starting the study interviews, from the ethical point of view, two aspects were assessed: the risk related to the analysis and use of the information data, as well as the potential emotional risks that the interview could cause to the participants (possible psychological destabilizations triggered by recalling grief and loss experiences of the past). If necessary, a quick and simple intervention aimed at restoring emotional balance, or even stopping, postponing or cancelling the interview, would have been carried out. It was also planned to offer counseling and/or withdraw the couple from the sample if at the time of starting the interviews any situation of emotional vulnerability had been detected. None of these situations occurred.

### 2.3 Procedure

An in-depth interview was conducted with all participating couples to examine their “life history” in order to obtain broad, extensive, and content-rich information and meanings about their parental experience.

The in-depth interview technique was chosen because it is an instrument that facilitates individualized and in-depth access to the life stories of the participating couples (Marin, 2021). This technique allowed to collect the subjective interpretations that the couples related from the lived experience.

The interviews were conducted in a context of privacy. The interviewer did not judge the contents of the answers to give couples greater freedom of communication.

All interviews were magnetophonically recorded and transcribed in their entirety. Their duration ranged from 60 to 90 min.

To segment the material into units of text, it was necessary to listen to the recordings several times in a row in order to be able to carry out that segmentation, depending on the conceptual framework and the objective of the study. It was done based on the criteria for indirect observation (Anguera, 2021; Krippendorf, 2013), choosing the syntactic criterion as it was considered the most appropriate.

From the segmentation of the text, 442 units of text were obtained, which were the citations selected as significant, resulting in a record of 249 codes. Once the coding was finished, a matrix of qualitative data was generated, whose columns were the respective subdimensions, and the rows were the units resulting from the segmentation.

The mixed methods approach followed in this research involves the three QUAL-QUAN-QUAL macro-stages (Anguera et al., 2020). The data collection corresponds to the first qualitative phase (QUAL), which has made it possible to obtain the code matrices from the observation instrument. Subsequently, the quantitative phase (QUAN) was carried out through quantitizing (Anguera, 2020; Arias-Pujol et al., 2022), in which the integration of qualitative and quantitative elements is materialized, taking as a reference the *via connect* proposed by Creswell and Plano Clark (2011, p. 7) within the framework of mixed methods. The code matrices are formed by qualitative data, but arranged in such a way (sequentially ordered rows of codes) that it has been possible to treat them quantitatively with respect to data quality control and data analysis.

### 2.4 Instruments

#### 2.4.1 Observation instrument

Based on the specific objectives of the study and previous experiences (Camps, 1992, 1996, 2010a, b, 2018), a flexible, indirect observation instrument *ad hoc* was designed that combines field format and category systems. The proposed dimensions are: (1) parenthood (process of child search until parenting); (2) infertility (Grief due to loss of fertility and coping with that grief); (3) communication/revelation of genetic origins; (4) affiliation (Psychological aspects of interpersonal relationships in the parenting experience in the family network); and (5) satisfaction of the experience (Perception of satisfaction from the positive to the negative degree).

Dimensions 1, 3, 4, and 5 were deployed in sub-dimensions, as indicated in Table 1, and these resulted in the construction of respective catalogues of conduct (non-exhaustive and mutually exclusive), while dimension 2 allowed the construction of a system of categories (exhaustive and mutually exclusive) (Anguera, 2003) (see Table 1).

**Table 1:** Indirect observation instrument *ad hoc* configured by field format combined with a category system: result of the deployment of dimensions 1, 3, 4, and 5 (which, in turn, allowed the creation of the respective behavior catalogs), and category system in dimension 2.

Dimensions	Instrument	Code	Subdimensions/category system
1. Parenting experience	FC	1A	Decision to have children
		1B	Decision ART
		1C	Pregnancy
		1D	Birth
		1E	Parenting
		1F	New families
2. Infertility experience	SC	2A	Loss of fertility emotions
		2B	Grief coping strategies
		2C	Coping with infertility during filiation
3. Genetic origins communication	FC	3A	Initial position revealing origins
		3B	Communication modality
		3C	Communication process
		3D	Who communicates
		3E	Agreed moment to communicate
		3F	Emotions in communication
		3G	To whom it is communicated
		3H	Reasons for communicating
		3I	Reasons for not communicating
4. Filiation value	FC	4A	Psychological value of the child
		4B	Corollary identifier
		4C	Perception of inter-family relationships
		4D	Perception of intergenerational relationships
		4E	Psychological aspects of the child
5. Filiation satisfaction	FC	5A	Positive experience
		5B	Difficult but positive experience
		5C	Negative experience

The five dimensions and sub-dimensions of the second, third, and fourth levels are defined in Table 2.

**Table 2:** Indirect observation instrument: definition of dimensions and sub-dimensions.

Dimension	Subdimensions
1. Parenting Positive and negative attitudes and emotional manifestations of the parenting process, from the moment of the decision to seek children until the parenting period	It is divided in six first-level sub-dimensions referring to the decision to have children and the use of ART, distinguishing the most biological aspects of parenthood, such as pregnancy and childbirth, from the more social and interpersonal aspects. Each of the sub-dimensions resulted in a behavioral catalogue.
2. Modality Of grief coping strategies (GCS) for infertility “grief of griefs” Emotional manifestations before the medical diagnosis of male factor infertility and the modality of grief coping strategies (GCS) for the loss of biological fertility.	It is divided in a System of three categories. 2A: Positive (2A1) and negative (2A2) emotional manifestations, generated by the medical diagnosis of infertility. 2B: Coping with Infertility Grief: Emotional reactions to the infertility diagnosis in terms of recognition/Acceptance or non-recognition/Denial of the difference between ART assisted fertility and biological fertility. 2B1 Acceptance of the loss of fertility: Couples who accept treatment with ART as a way to conceive children, recognizing the difference between assisted fertility and biological fertility and the emotional effects generated by the loss of fertility. 2B2 Denial of the loss of fertility: Couples who do not recognize any difference between assisted fertility and biological fertility, establishing a total equivalence between them. They accept treatment with ART attributing a replacement value to biological fertility. 2B3 Reaction to depressive adjustment: Couples who have a mild, moderate or severe anxious-depressive state, according to the DSM-V classification, reactive to the medical diagnosis of infertility. 2C Coping with grief during parenting: communication style used by the couple in interpersonal relationships with children and family during the parenting period according to recognition/Acceptance or Non-recognition/Denial of infertility. 2C1 Active coping strategies: Couples who incorporate in their relationship with their children and family the communication of the infertility experience, actively and voluntarily promoting communication about the medical treatment received. They use various adaptive confrontational psychological resources depending on when the communication occurs. They use a coping system that allows situations of external reality to be introduced to evoke the recognition of the loss of fertility and/or communication of genetic origins, even if they cause emotional pain. 2C2 Passive coping strategies: Couples who voluntarily or involuntarily inhibit attitudes of active search for the recognition and/or communication of genetic origins. 2C3 Avoidant coping strategies: Couples who actively and voluntarily use strategies to cancel the possibility of the infertility problem being present in communication with children and family. They present defensive psychological mechanisms such as denial, avoidant excuses or control over potential trigger situations of questions on the subject in the service of not addressing the problem.
3. Genetic origins communication Attitudes adopted from before birth of the child for the decision to share or keep the information secret. Modalities of communication used and emotional manifestations lived according to the concrete experience carried out.	It is divided in nine sub-dimensions that refer to the psychological aspects of the entire process related to the modality, time and people to whom it is wanted to inform, about the decision to reveal or not the genetic origins to the child and/or the community. Each of the sub-dimensions resulted in a behavioral catalogue
4. Filiation positive and negative attitudes and emotional manifestations related to the filiation process that describe the value that parents place on the child and parenting in their own family system and in the extended family; as well as in the management of inter-family relationships and information about the genetic origins of the child and the social-family environment.	It is divided in five first-level sub-dimensions that describe the psychological value that the child has for them in their life project, aspects and psychological problems of the child and the emotional management of inter-family relationships. Each of the sub-dimensions resulted in a behavioral catalogue
5. Satisfaction of parenting experience Describes the experience of satisfaction that the couple expresses over the years, in relation to the parental journey of raising children.	It is divided in three first-level sub-dimensions that describe, on a scale defined between positive experience, difficult but positive experience and negative experience, the experience of gratification expressed by the couple in cognitive and affective terms in relation to the upbringing of the children. Each of the sub-dimensions resulted in a behavioral catalogue.

#### 2.4.2 Recording instruments and software

All interviews were recorded with the Olympus recorder model WS-560 M.

The Atlas.ti software version 7.5.17. was used for the coding of the record.

The free program GSEQ allowed data quality control and lag sequential analysis to be carried out^1^ (Bakeman and Quera, 2011).

### 2.5 Data quality control

The evaluation of data quality control was performed by looking for inter-observer agreement using Cohen’s Kappa statistic (Cohen, 1960, 1968). It was carried out based on the coding by a second researcher (Anguera, 2003), of 15% of the totality of the interviews. The agreement was calculated with the GSEQ software. According to the criteria of Landis and Koch (1977), the level of agreement was almost perfect with an average of 0.856 kappa values.

### 2.6 Data analysis

In this study, a lag sequential analysis is carried out for all first-level dimensions and sub-dimensions of all the couples in the sample in order to establish associations between the interactive communication episodes produced from lags −1 (retrospectively) to +3 (prospectively) (Bakeman and Quera, 2011). Although we made calculations with a greater number of lags, it did not provide relevant information.

### 2.7 Multiple case detection

Over the last three decades, the resolution of multiple situations about which there are systematic records on different “cases” has been extensively investigated, whether they are participants in a study, members of a group, interlocutors of the same *partner*, or couples, as in our study.

While the single case can describe a certain phenomenon in a rich and nuanced way (Siggelkow, 2007), the multiple case (Yin, 2014) offers a solid basis for the construction of the theory, activating the possibility of discriminating between the idiosyncratic and the consistently replicated; in addition, the multiple case allows reaching a degree of abstraction that will favor its robustness. Another question will be how to elucidate the existence of a uniform or common structure, in whole or in part, in the multiple case, and from the aggregation of particular cases (Anguera, 2018).

In this study we will propose multiple cases based on the results obtained through the lag sequential analysis, materializing the second qualitative macro-stage (QUAL) in accordance with the approach *mixed methods* indicated.

## 3 Results

### 3.1 Behavior patterns

In the lag sequential analysis, each of the subdimensions was proposed as criterion behavior and all subdimensions as conditioned behavior. The total number of behavior patterns was 55.

We highlight that all couples without exception generate one or more patterns of behavior in dimensions 1 and 4; in dimension 2, all but two; in dimension 3, all but one and in dimension 5 all but three.

**Figure 1:**
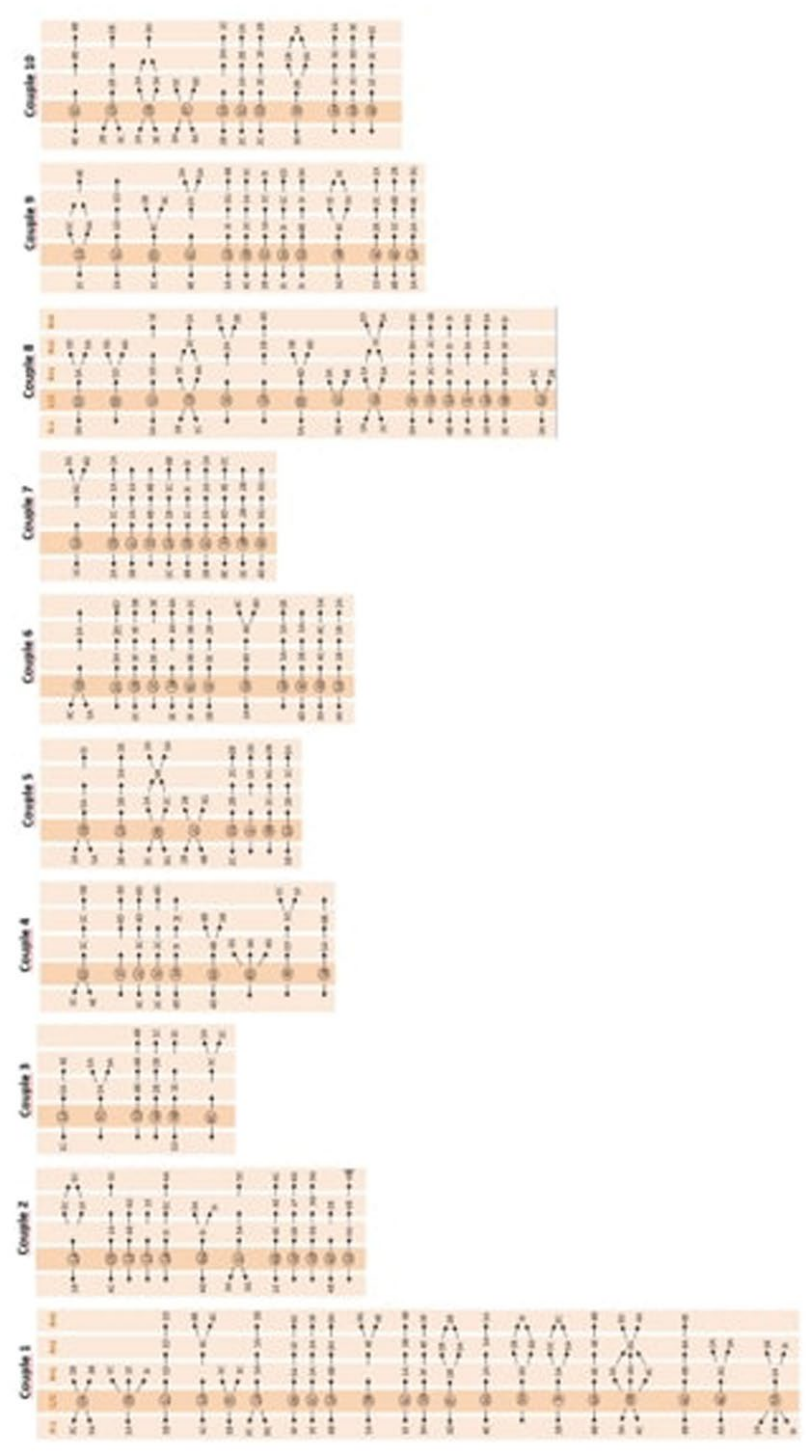
Patterns of behavior of all couples.

The behavior patterns of the 10 couples are included in Figure 1.

Table 3 presents verbatim excerpts from interviews with parents who conceived through donor insemination. Dimension 2B refers to modalities of grief elaboration, while dimensions 3A and 3B relate to communication practices concerning genetic origins (see Table 3).

**Table 3:** Examples of grief processing and communication of genetic origins in donor conception families.

Dimension	Subdimension	Example 1	Example 2
2B Grief Coping Strategies (GCS)	Acceptance of the biological loss (2B1)	“Neither of us has to reproach anything… We complement each other… we have been married for 27 years…”	“What can you think… it is what it is… you cannot do anything else.”
	Denial/non-recognition (2B2)	“I… think we have lived through it… it has not been a topic of conversation, as if it never happened.”	“There is no difference, I will still be the father…”
	Third-Party Reproduction as equivalent/substitute for biology (2B22)	“It does not matter, what counts is that we’ll be able to have a child…”	“The one who was waiting was both of us… sperm is simply a small thing…”
	Active avoidance (during child-rearing) (2C1)	“We have not talked about it anymore, we already have the kids…”	“At home it’s a closed subject, we have not spoken about it further. It’s not a secret, but there’s no need.”
	Confrontation to give meaning (2C3)	“Do you think we should not tell…”	“It has not been a taboo subject, we have talked a lot about everything.”
3A Communication of genetic origins	Agreement to disclose (3A1)	“We always had it clear from the beginning… just like if we had adopted.”	“We thought about it that way from the start, and we did it as we planned, without changing our mind.”
	Agreement not to disclose (3A2)	“From the beginning, we were very clear that he/she should not know… and up to today…”	“Your decision has to be respected, you are more comfortable if he/she does not know.”
	Progressive modality (3B1)	“Since childhood, like a story… we told him/her there were different ways of being born…”	“We always tried to explain it little by little so she could understand…”
	Direct modality (3B2)	“One day I just told him and he said: ‘ah, okay, I already knew there was something’…”	“I told him: ‘Oriol, we had insemination with donor semen…’ He answered: ‘ah!! okay, it does not matter.’”
	Mixed modality (3B3)	“Later I told him: you were born differently… dad had problems and went to the doctors…”	“Dad had problems and they operated on him, and mom was then able to have you…”
	Fear of child’s reaction (3F1)	“My only problem… is that if one day he finds out… that really makes me anxious.”	“If he found out now, he would feel bad that we had not told him before.”

In particular, the idiographic study of each couple is described below to show the association between the criterion behaviors with all significant conditioned behaviors (see Table 4).

**Table 4:** Association between the criterion behaviors with all significant conditioned behaviors for each couple.

Couples	Criterion behavior and conditioned behaviors
Couple 1	GCS behaviors, with the process of communicating genetic origins, with the experience of filiation and this with parental satisfaction
Couple 2	Behaviors of the communication process of genetic origins with the experiences of the filiation experience and with negative parental satisfaction
Couple 3	Parental satisfaction behaviors with the decision to seek children and with the identification corollary of their children
Couples 4–10	GCS behaviors, with the process of communicating genetic origins, with the experience of filiation and this with parental satisfaction

### 3.2 Multiple cases

Five multiple cases have been obtained from the selection of patterns that show the presence of regularities in all couples, extracted from the results obtained through the lag sequential analysis, and that are related to different dimensions of the observation instrument (see Table 5).

**Table 5:** Selection of behavior patterns of all couples.

Couple 1	Couple 2	Couple 3	Couple 4	Couple 5
1A 5A / 1B 1A 3C / 3B 2A 3G / 5A 1A 2A 5A / 1A 1B 3G 4E / 2A 4A 4B / 4E 4B 4C / 4A	1A 1B / 5C 3G 3I / 3A 3A 3I 1E 4B 4E 4B 4D 3G 4C 1B 5C / 4A	1A 1C / 5A 1D 4B	1D 4E / 1C 2C 3C / 4D 3G 4D 5B 4A /4B	1B 2A 2B/ 5A 2B 2C 2B 3G / 2C 2C 4B / 3G

Couple 6	Couple 7	Couple 8	Couple 9	Couple 10
4A 5A 4C 1B / 2A 2A 3H 4D / 3H 4C 2C 2B / 3F 3E 3B 2B 3F / 3E	1B 1C / 1A 1B 2A / 1C 2B 4B 3G 4E / 4D	1A 3A / 5A 3A 5A / 1C 1C / 1D –5A 1B 1D / 4A 2C 3G / 4B 3H 4B / 3F 3I 3F 3H / 3I	1A 2C / 1C 2B 4C / 2C 1A 1A 5A / 2A 4E 4B 4E / 1E 1D 4C / 2B 3G / 4B – 3I	2A 3C / 2B 2B 3E / 3A 2B 3A / 3H 2C 3H / 3C 2C 4A / 3G 3C / 2C 2A 3G / 3E – 2C 3G 3E / 2B 4E 1E

#### 3.2.1 Multiple case I: behavior patterns related to the parenting (D1)/filiation satisfaction (D5) dimensions

Results show that seven couples (P1, P2, P3, P5, P6, P8, and P9) show regularities in the pattern of behavior that links the experiences related to the access to parenthood, in particular those associated with the decision to have children and the use of reproductive techniques with donor semen, with the experience of the satisfaction finally obtained in their experience of filiation (see Table 6).

**Table 6:** Multiple case I: relationship between access to parenthood and filiation satisfaction.

Couple 1	Couple 2	Couple 3	Couple 4	Couple 5
**1A 5A / 1B** 1A 3C / 3B **2A 3G / 5A 1A** **2A 5A / 1A 1B** 3G 4E / 2A 4A 4B / 4E 4B 4C / 4A	**1A 1B / 5C** 3G 3I / 3A 3A 3I 1E 4B 4E 4B 4D 3G 4C 1B **5C / 4A**	**1A 1C/ 5A** 1D 4B	1D 4E / 1C 2C 3C / 4D 3G 4D 5B 4A /4B	**1B 2A 2B/ 5A** 2B 2C 2B 3G / 2C 2C 4B / 3G

Couple 6	Couple 7	Couple 8	Couple 9	Couple 10
**4A 5A 4C 1B / 2A** 2A 3H 4D / 3H 4C 2C 2B / 3F 3E 3B 2B 3F / 3E	1B 1C / 1A 1B 2A / 1C 2B 4B 3G 4E / 4D	1A 3A / 5A 3A 5A / 1C **1C / 1D – 5A** 1B 1D / 4A 2C 3G / 4B 3H 4B / 3F 3I 3F 3H / 3I	1A 2C / 1C 2B 4C / 2C 1A **1A 5A / 2A 4E** 4B 4E / 1E 1D 4C / 2B 3G / 4B – 3I	2A 3C / 2B 2B 3E / 3A 2B 3A / 3H 2C 3H / 3C 2C 4A / 3G 3C / 2C 2A 3G / 3E – 2C 3G 3E / 2B 4E 1E

The patterns of behavior manifested by the existing regularities have been highlighted in bold.

Seven of the participating couples establish a relationship between the decision to have children and/or use AID with the experience of satisfaction achieved. Of all of them, six describe an experience of positive satisfaction (P1, P3, P5, P6, P8, and P9), but one (P2) describes an experience of negative satisfaction, expressing that it should not have done so; however, four couples (P1, P5, P6, and P9) of those who describe positive satisfaction, also articulate the pattern with the modality of coping with the grief for losing fertility (GCS).

In summary, we highlight that in the set of stories of the couples who present this multiple patterns, six couples report that the decision to use AID to face infertility has helped them to be able to conceive children and carry out a satisfactory parental experience; in addition, four of these couples, in the sequence of their story, also relate this experience to the grief that infertility meant for them. The P2 couple (separated mother) who reports an experience of negative satisfaction stands out, in whose story there are no associations related to infertility grief. We also highlight that there are three couples from all the participants who in their story have not significantly associated access to parenthood with the assessment of a satisfying experience.

#### 3.2.2 Multiple case II: behavior patterns related to dimensions GCS (D2)/communication of origins (D3)/filiation (D4)

Six of the participating couples (P1, P4, P5, P6, P8, and P10) show regularities in the pattern of behavior that describes that grief coping strategies (GCS) are related by prospective and/or retrospective alternation with communication of genetic origins and filiation experience (see Table 7).

**Table 7:** Multiple case II: relationship between grief coping strategies (GCS), communication of origins and experience of filiation.

Couple 1	Couple 2	Couple 3	Couple 4	Couple 5
1A 5A / 1B 1A 3C / 3B **2A 3G / 5A 1A** 2A 5A / 1A 1B **3G 4E / 2A** 4A 4B / 4E 4B 4C / 4A	1A 1B / 5C 3G 3I / 3A 3A 3I 1E 4B 4E 4B 4D 3G 4C 1B 5C / 4A	1A 1C/ 5A 1D 4B	1D 4E / 1C **2C 3C / 4D** 3G 4D 5B 4A /4B	1B 2A 2B/ 5A 2B 2C **2B 3G / 2C** **2C 4B / 3G**

Couple 6	Couple 7	Couple 8	Couple 9	Couple 10
4A 5A 4C 1B / 2A **2A 3H 4D / 3H 4C** **2C 2B / 3F 3E 3B** **2B 3F / 3E**	1B 1C / 1A 1B 2A / 1C 2B 4B 3G 4E / 4D	1A 3A / 5A 3A 5A / 1C 1C / 1D –5A 1B 1D / 4A **2C 3G / 4B** 3H 4B / 3F 3I 3F 3H / 3I	1A 2C / 1C 2B 4C / 2C 1A 1A 5A / 2A 4E 4B 4E / 1E 1D 4C / 2B 3G / 4B – 3I	**2A 3C / 2B** **2B 3E / 3A** **2B 3A / 3H** **2C 3H / 3C** **2C 4A / 3G** **3C / 2C 2A** **3G / 3E – 2C** **3G 3E / 2B** 4E 1E

The patterns of behavior manifested by the existing regularities have been highlighted in bold.

The pattern describes that, in the account of these six couples, in relation to the GCS, although they express that the emotions emerging from the infertility diagnosis are negative, only three present communications associated with the behavior related to the recognition or not of the loss of fertility (P5, P6, and P10), on the other hand, five of them (P4, P5, P6 P8, and P10) relate it to the parenting process.

#### 3.2.3 Multiple case III: behavior patterns related to dimensions GCS (D2)/parentality (D1)

The results show that five of the participating couples (P1, P5, P6, P7, and P9) present regularities in the behavior patterns that describe that the behavior related to the GCS are articulated through prospective and/or retrospective alternation with the decision to seek children and/or use AID. We will highlight that, in this multiple pattern, the behaviors that describe the emotions emerging from the infertility diagnosis related to GCS are always negative (see Table 8).

**Table 8:** Multiple case III: relationship between grief coping strategies (GCS) and process of access to parenthood.

Couple 1	Couple 2	Couple 3	Couple 4	Couple 5
1A 5A / 1B 1A 3C / 3B **2A 3G / 5A 1A** **2A 5A / 1A 1B** **3G 4E / 2A** 4A 4B / 4E 4B 4C / 4A	1A 1B / 5C 3G 3I / 3A 3A 3I 1E 4B 4E 4B 4D 3G 4C 1B 5C / 4A	1A 1C/ 5A 1D 4B	1D 4E / 1C 2C 3C / 4D 3G 4D 5B 4A /4B	**1B 2A 2B/ 5A** 2B 2C 2B 3G / 2C 2C 4B / 3G

Couple 6	Couple 7	Couple 8	Couple 9	Couple 10
**4A 5A 4C 1B / 2A** **2A 3H 4D / 3H 4C** 2C 2B / 3F 3E 3B 2B 3F / 3E	1B 1C / 1A **1B 2A / 1C** 2B 4B 3G 4E / 4D	1A 3A / 5A 3A 5A / 1C 1C / 1D –5A 1B 1D / 4A 2C 3G / 4B 3H 4B / 3F 3I 3F 3H / 3I	1A 2C / 1C 2B 4C / 2C 1A **1A 5A / 2A 4E** 4B 4E / 1E 1D 4C / 2B 3G / 4B – 3I	2A 3C / 2B 2B 3E / 3A 2B 3A / 3H 2C 3H / 3C 2C 4A / 3G 3C / 2C 2A 3G / 3E – 2C 3G 3E / 2B 4E 1E

The patterns of behavior manifested by the existing regularities have been highlighted in bold.

#### 3.2.4 Multiple case IV: behavior patterns related to dimensions infertility (D2)/filiation (D4)

The results show that eight of the participating couples (P1, P4, P5, P6, P7, P8, P9, and P10) have regularities in behavior patterns that describe that GCS-related behavior is associated by prospective and/or retrospective alternation with filiation experience. Of these couples, six (P1, P4, P5, P6, P8, and P10) also associate the pattern with communication of origins and four (P1, P5, P8, and P10) specifically with the behavior to which genetic origins are communicated (see Table 9).

**Table 9:** Multiple case IV: relationship between grief coping strategies (GCS) and filiation experience.

Couple 1	Couple 2	Couple 3	Couple 4	Couple 5
1A 5A / 1B 1A 3C / 3B 2A 3G / 5A 1A 2A 5A / 1A 1B **3G 4E / 2A** 4A 4B / 4E 4B 4C / 4A	1A 1B / 5C 3G 3I / 3A 3A 3I 1E 4B 4E 4B 4D 3G 4C 1B 5C / 4A	1A 1C/ 5A 1D 4B	1D 4E / 1C **2C 3C / 4D** 3G 4D 5B 4A /4B	1B 2A 2B/ 5A 2B 2C 2B 3G / 2C **2C 4B / 3G**

Couple 6	Couple 7	Couple 8	Couple 9	Couple 10
**4A 5A 4C 1B / 2A** **2A 3H 4D / 3H 4C** 2C 2B / 3F 3E 3B 2B 3F / 3E	1B 1C / 1A 1B 2A / 1C **2B 4B** 3G 4E / 4D	1A 3A / 5A 3A 5A / 1C 1C / 1D –5A 1B 1D / 4A **2C 3G / 4B** 3H 4B / 3F 3I 3F 3H / 3I	1A 2C / 1C 2B 4C / 2C 1A 1A 5A / 2A 4E 4B 4E / 1E **1D 4C / 2B** 3G / 4B – 3I	2A 3C / 2B 2B 3E / 3A 2B 3A / 3H 2C 3H / 3C **2C 4A / 3G** 3C / 2C 2A 3G / 3E – 2C 3G 3E / 2B 4E 1E

The patterns of behavior manifested by the existing regularities have been highlighted in bold.

#### 3.2.5 Multiple case V: patterns of conduct related to the dimensions of origin communication (D3)/filiation (D4)

The results show that nine of the participating couples (P1, P2, P4, P5, P6, P7, P8, P9, and P10) present regularities in behavior patterns that describe that behavior related to the communication of genetic origins is related through prospective and/or retrospective alternation with filiation experience. In relation to the communication of genetic origins, we underline that seven of them refer particularly to the person who communicates (see Table 10).

**Table 10:** Multiple case V: relationship between communication of origins and filiation experience.

Couple 1	Couple 2	Couple 3	Couple 4	Couple 5
1A 5A / 1B 1A 3C / 3B 2A 3G / 5A 1A 2A 5A / 1A 1B **3G 4E / 2A** 4A 4B / 4E 4B 4C / 4A	1A 1B / 5C 3G 3I / 3A 3A 3I 1E 4B 4E 4B **4D 3G** 4C 1B 5C / 4A	1A 1C/ 5A 1D 4B	1D 4E / 1C **2C 3C / 4D** **3G 4D** 5B 4A /4B	1B 2A 2B/ 5A 2B 2C 2B 3G / 2C **2C 4B / 3G**

Couple 6	Couple 7	Couple 8	Couple 9	Couple 10
4A 5A 4C 1B / 2A **2A 3H 4D / 3H 4C** 2C 2B / 3F 3E 3B 2B 3F / 3E	1B 1C / 1A 1B 2A / 1C 2B 4B **3G 4E / 4D**	1A 3A / 5A 3A 5A / 1C 1C / 1D –5A 1B 1D / 4A **2C 3G / 4B** **3H 4B / 3F 3I** 3F 3H / 3I	1A 2C / 1C 2B 4C / 2C 1A 1A 5A / 2A 4E 4B 4E / 1E 1D 4C / 2B **3G / 4B – 3I**	2A 3C / 2B 2B 3E / 3A 2B 3A / 3H 2C 3H / 3C **2C 4A / 3G** 3C / 2C 2A 3G / 3E – 2C 3G 3E / 2B 4E 1E

The patterns of behavior manifested by the existing regularities have been highlighted in bold.

## 4 Discussion

We have obtained relevant results in the methodological field, which shows the potential and suitability of the *mixed methods* that allow quantifying a highly complex experience such as filiation through assisted reproduction from adequately structured qualitative information (Anguera, 2018; Stake, 2006; Yin, 2014). The in-depth treatment of the interview from the *mixed methods* perspective has allowed us to transform the observed data from a very difficult to quantify experience, into quantitative results rich in qualitative nuances, on the experience studied. At present, there is still little research available with *mixed methods* in this field (Sandelowski, 1988; Sandelowski et al., 2009).

Anguera (2021) highlights the innovations made on the multimodal perspective of communication applied to the in-depth interview and points out that the transcription of interviews, highly systematized from a more or less molarized or molecularized coding, makes it possible to obtain matrices of codes that, although they contain qualitative information, the degree of systematization makes them already suitable for quantitative analysis. Along the lines described by the author, in our study we have applied this procedure to detect the structure of significant associations in the behaviors/communications of the couples reports; and so we have mapped the interrelationships between the codes/categories that have given us the results of the study.

In line with the mixed-methods approach adopted, neither sample size nor generalizability is a primary concern. The study aims, rather, to generate in-depth insights and enhance understanding of the phenomenon explored.

The results describe five multiple cases based on behavioral patterns that show that the participating couples, in their report, establish a relationship of association between the behaviors related to the elaboration of the grief from the loss of fertility and the communication of the genetic origins; and they show that 25 years after conceiving children, the two behaviors are still significantly connected to each other.

The detailed interpretation of each case provides very valuable information to determine in detail specific aspects of the experience studied on the grief of infertility and that are essential for its in-depth analysis.

*Multiple case I* shows that the experience of loss caused by infertility lasts over time and that its psychological effects, as highlighted by other authors (Hammarberg et al., 2008), fall on filiation, from the experience of pregnancy, childbirth, to the bonds of attachment established in upbringing.

This result allows us to affirm the emotional value of the loss of fertility by couples and its significance in the upbringing of their children. For this reason, we find it necessary to consider the recognition and acceptance of the loss of biological fertility as a psychological factor to be taken into account in the first line of the fertility clinic when the diagnostic process is carried out.

*Multiple case II* yields results along the decision to communicate or not to communicate genetic origins; and if so; how, when and what modality to do it. There are highly polarized voices against and in favor of communication, but most authors agree that the decision of whether or not to communicate genetic origins is a delicate issue that couples do not know how to deal with (Blake et al., 2010; Daniels and Thorn, 2001; Kirkman, 2003; MacDougall et al., 2007).

In the case of two-parent heterosexual couples, clear difficulties have been observed in making this decision. Our clinical practice agrees with Fitó (2010) that the problem of the decision to communicate origins cannot be solved simply by offering information.

The results of this study allow us to affirm that the decision to reveal one’s genetic origins to one’s children is related to the experience of loss experienced by the diagnosis of infertility.

*Multiple case II* (which establishes a clear relationship between the dimensions of grief coping strategies (GCS) and communication of origins) reinforces the clinical indication of favoring *a priori* the recognition of the loss of fertility and the processing of the grief that it entails, so as to facilitate the subsequent management of decision-making on the communication of genetic origins, according to the singularities chosen by each couple.

The application of this result in the fertility clinic is crucial, in particular, with regard to the orientation of the interventions that can be offered by physicians and professionals in the first line of consultation.

From the perspective of the first-line care approach, we believe that the counseling function carried out by specialists has a very important educational and preventive role in the adjustment of the dynamics of couples in the upbringing. Kazemi et al. (2021) also highlight the need for mental health programs to improve adaptive coping strategies of men undergoing ART treatment. Likewise, Luca et al. (2021) propose to consider a couple-centered program for the integrated management of psychological and sexual dysfunction in ART programs.

Along these lines, the articulation of the results of multiple case II with those of multiple case I highlight the importance of orienting care objectives to the improvement of parental functions and not only to increase the pregnancy rate.

*Multiple case III* shows that the therapeutic offer of assisted reproduction techniques, quickly proposed, can have effects on coping with the grieving process, obstructing or facilitating the reparative function.

*Multiple case IV* shows that, 25 years after treatment, the parent couples continue to associate the emotional experience of the loss of fertility with the decision to reveal the genetic origins and values of the experience of filiation. This multiple case is related to *multiple case II*, that allow us to highlight again the clinical importance of coping with and processing infertility grief (GCS).

*Multiple case V* shows the influence that emotional management has had when incorporating the experience of sperm donation in interpersonal ties with the child and family.

The integrated reading of the *multiple cases II, IV, and V*, in relation to the objectives of the study, shows a significant associative relationship that relates the emotions emerging from the loss of fertility and coping with grief, and the decision to reveal the genetic origins.

This study presents several limitations that should be acknowledged. Firstly, the 25-year interval between treatment and follow-up may have introduced memory biases among the interviewed participants. Retrospectively constructed narratives—particularly those concerning initial decisions about origin disclosure and the subjective elaboration of infertility grief—may be selectively recalled, reconstructed, or aligned with current family narratives.

Although a sequential mixed-methods design (QUAL–QUAN–QUAL) and rigorous coding procedures were employed, such recall effects cannot be entirely ruled out. Nevertheless, considering that the study focuses on grief coping, a retrospective perspective is particularly valuable for understanding how couples have re-signified their experiences over time.

Self-selection bias should also be considered, as only couples willing to be interviewed chose to participate, possibly representing more resilient or motivated cases. In addition, social desirability may have influenced how participants described their parental experiences. The absence of systematic measures of psychological functioning at both baseline and follow-up limits the emotional contextualization of the findings.

Furthermore, the study is culturally situated in Spain, with recruitment conducted within a specific clinical context in Barcelona. Cultural scripts related to reproduction, kinship, and disclosure—as well as the structure of public reproductive services—are highly specific to this setting and may limit the transferability of findings.

Future research would benefit from incorporating the voices of sons and daughters, thereby including the perspective of the offspring. Additionally, conducting similar studies with couples who conceived without donation, single-parent families, lesbian couples, or those formed through other assisted reproduction pathways would offer valuable comparative insights.

The application of these results in the healthcare field strongly supports the clinical recommendation to facilitate the recognition of fertility loss and grief in infertile couples to promote healthier parenting roles (Tavousi et al., 2022). More specifically, in light of the findings of this study, we propose the following practical clinical applications: (1) To promote interdisciplinary collaboration across all reproductive units, whether public or private, by incorporating mental health professionals with specialized training in fertility-related clinical practice. (2) To develop care protocols that, from the initial consultations, include an assessment of the emotional dimensions of fertility and the parental project that leads to the birth of children. (3) To incorporate the semi-structured interview as the primary assessment tool, complemented where necessary by psychodiagnostic scales and/or instruments to detect emotional needs and guide interventions. (4) To include in clinical protocols the design of psychosocial interventions aimed at fostering parental well-being and strengthening the resilience of parent–child bonds, with particular attention to grief processing in cases of genetic donation and to cultural differences in kinship conceptions and diverse family trajectories.

## Data Availability

The raw data supporting the conclusions of this article will be made available by the authors, without undue reservation.

## Footnotes

^1^ https://www.mangold-international.com/en/products/software/gseq.html

## In Memoriam

We dedicate this work to the memory of Professor M. Teresa Anguera, whose intellectual rigor, generosity, and pioneering contributions to observational methodology continue to inspire our research and teaching. Her legacy endures in every project that seeks to understand human behavior with depth and precision.
